# Arbovirus circulation among febrile patients at the greater Accra Regional Hospital, Ghana

**DOI:** 10.1186/s13104-019-4378-x

**Published:** 2019-06-11

**Authors:** Simon Kofi Manu, Joseph Humphrey Kofi Bonney, Deborah Pratt, Farida Njelba Abdulai, Eudosia Esinam Agbosu, Prince Osei Frimpong, Theophilus Korku Adiku

**Affiliations:** 1 Department of Microbiology, University of Ghana Medical School, College of Health Sciences, University of Ghana, Legon, Accra, Ghana; 2 Department of Virology, Noguchi Memorial Institute for Medical Research, College of Health Sciences, University of Ghana, Legon, P.O. Box LG 581, Accra, Ghana; 3 Department of Biomedical Sciences, University of Health and Allied Sciences, Ho, Ghana; 4 Public Health Department, Greater Accra Regional Hospital, Ridge, Accra, Ghana

**Keywords:** Dengue, Chikungunya, Polymerase chain reaction, Differential diagnosis

## Abstract

**Objective:**

Arboviruses, Dengue and Chikungunya have become major international public health concerns due to their epidemics and introduction in new areas. In Ghana, little is known is about Dengue and Chikungunya viruses though the country has been listed as part of the 34 countries in which the viruses are endemic. This has been attributed partly to the lack of diagnostic tools for these viruses in several health facilities and institutions across the country. The purpose of this study was to detect and characterize these viral pathogens among febrile patients in Accra Ghana.

**Results:**

This hospital-based cross-sectional study enrolled 260 suspected Dengue and/or Chikungunya febrile patients who submitted their clinical specimens of serum. Out of the total number tested with both molecular and serological tools, Chikungunya and Dengue specific total antibodies were detected from 72 (27.69%) and 180 (69.23%) respectively. None of the participants tested positive for Dengue and Chikungunya by reverse transcription-polymerase chain reaction (RT-PCR) and with the Dengue-specific NS1 antigen strip kits. Our findings suggested that Dengue and Chikungunya viruses may be circulating but are being missed among febrile patients. Differential diagnosis work-up in febrile patients should be made to include Dengue and Chikungunya infections.

## Introduction

Arbovirus is an acronym for “Arthropod borne virus” and is used to refer to any of the viral pathogens including Dengue fever and Chikungunya viruses (DENV and CHIKV) that are transmitted by arthropod vectors [1]. Arboviruses affect both animals and plants and in humans, symptoms of infection generally occur 3–15 days after virus exposure and last 3 or 4 days. The most common clinical features of infection are fever, headache, and malaise, but encephalitis and hemorrhagic fever may also occur [2]. Dengue viral infections have become major international public health concern due to their epidemics and introduction in new areas. The virus is the most rapidly spreading and frequently encountered mosquito-borne viral infection with increased incidence of 30-fold and explosive outbreaks over the last decade [3]. Currently the virus is endemic in more than 100 countries in the WHO regions of Africa, the East Mediterranean, South East Asia and Western Pacific [2]. A global annual estimate of 50 to 100 million cases of Dengue fever in endemic areas have been documented to account for 250,000 to 500,000 cases of Dengue hemorrhagic fever with 20,000 deaths and 264 disability adjusted life years per million population per year are lost [4].

Chikungunya has been reported from over 60 countries in Africa, Asia, Europe and the Americas. After its detection in 1953 from Africa, the virus has been circulating at relatively low levels in Africa until 2000 where large outbreaks occurred in Democratic Republic of Congo, Gabon and Kenya [5].

Transmission of DENV and CHIKV have been reported endemic in 34 countries in the African regions [6]. They have also been diagnosed in travelers from Europe and North America returning from several West Africa countries including Ghana [7, 8]. Though the burden has increased for both viral infections, disease epidemiology in the African region have not been well documented.

In the sub-region many febrile cases are presumptively diagnosed and treated for malaria. This is partly due to lack of diagnostic tools for these viruses in health facilities. Enormous attention given to the burden of malaria in Africa has been to the detriment of other febrile-causing pathogens including DENV and CHIKV [9]. This study is intended to augment limited data in the sub-region and provide a basis for the establishment of a surveillance program to minimize spread and possibly avert epidemics.

## Main text

Dengue and Chikungunya viruses share a common vector for transmission, mostly co-infect and their infections are characterized by overlapping clinical signs. In most affected areas where the DENV is now found, it has been established that all the four virus serotypes are circulating [10]. The CHIKV with three different genotypes: the western African, the east-central-south African (ECSA), and the Asian genotype [11], initially restricted to Central Africa and Asia, has recently invaded new territories. Although *Aedes aegypti* is the main vector of both viruses, *Aedes albopictus* has been documented lately to be playing an important role in contributing to the spread in temperate climate areas [12]. Here we sought to detect and characterize DENV and CHIKV from patients to establish the circulating viruses which hitherto have had little diagnostic attention.

### Methods

#### Study site and subjects

Hospital based cross sectional study conducted between May 2016 to April 2017 at the Greater Accra Regional Hospital—a 470-bed capacity tertiary facility which provides a wide range of general and specialists services. The study population were patients of all ages who sought medical care and consented to be part.

#### Eligibility criteria

Consented subjects were enrolled as suspected Dengue cases when diagnosed with acute febrile illness (> 38.5 °C) ongoing within 5 days and with two or more of the following clinical manifestations; headache, retro-orbital pain, leucopenia, myalgia, arthralgia, rash, hemorrhagic manifestations.

Suspected Chikungunya cases were defined as an acute febrile illness (> 38.5 °C) and severe arthralgia not explained by malaria or any other medical conditions and having reported transmission within 15 days. Criteria for exclusion included patients who declined to be part of the study and/or refuse to submit samples after consent. Acute cases were confirmed as such by RT-PCR test assay.

#### Sample and data collection

Structured questionnaires were administered for socio-demographic and clinical data from consented subjects and a maximum of 5 ml whole blood collected into an anticoagulant-free test tube. The samples were processed into serum and aliquoted into 2 separate vials of 1 ml each and kept temporarily at − 20 °C.

#### Testing assays

Serum specimens were tested with Dengue NS1 antigen strip, IgG and IgM capture ELISA assay and RT-PCR assay for both Dengue and Chikungunya viruses.

#### Reverse transcription-polymerase chain reaction

Nucleic acid was extracted and purified from the serum with QIAamp Viral Mini Extraction kit (Qiagen, Hilden, Germany); and a total of 60 μl purified RNA was labelled and cryopreserved. The pre-PCR reaction mixture was prepared with AgPath-ID™ One Step RT-PCR kit (Applied Biosystems, California USA) and Trioplex Real-time RT-PCR kit (CDC; catalog #KT0166) by the US CDC and designed to qualitatively detect and differentiate RNA from Zika, Dengue, and Chikungunya virus. To test the purified extracts by RT-PCR, instructions from the kit’s manufacturers were followed and after-run analysis were performed separately for each target.

#### Serology test

Serum samples were tested for specific Dengue and Chikungunya IgM and IgG antibodies using the Anti-Dengue Virus IgM and IgG ELISA kits (Abcam, Cambridge, MA, USA) and Anti- Chikungunya Virus IgM and IgG ELISA kits (Abcam, Cambridge, MA, USA). The test procedures were done in accordance with manufacturer’s instructions.

#### Dengue NS1 antigen assay

All the samples were also qualitatively detected for the Dengue virus NS 1 antigen using the Dengue NS 1 Ag strip (Bio-Rad, Marnes-la-Coquette, France). The test was done in accordance with manufacturer’s instruction.

#### Data analysis

Generated data were analyzed with Statistical Package for Social Sciences (SPSS) version 22. Univariate analysis including frequencies and measures of tendency were performed to show the fraction of infection and exposure among subjects. Categorical variables were expressed in the form of percentages and frequencies. Chi square tests were used to determine significant differences in categorical variables and *p* values ≤ 0.05 were considered significant.

### Results

#### Demographics/clinical presentation

The study subjects included 84 (32%) males and 176 (68%) females with the highest proportion, 155 (60%) within the age group of 21 to 40 years (Table 2).

Frequently reported symptoms other than fever were loss of appetite (92%), joint pain (86%) and muscle pain (85%). Others were diarrhea, extreme weakness after rehydration, nausea, vomiting, conjunctivitis, chest pains, rapid respiration and recent loss of hearing as shown in Table 1. Four of the participants (2%) reported hemorrhagic manifestations.

**Table 1:** Clinical presentation of study subjects

Variables	Frequency	Percent
Nausea	115	44
Muscle pain	222	85
Joint pain	223	86
Diarrhea	148	57
Extreme weakness after rehydration	44	17
Vomiting	105	40
Loss of appetite	239	92
Conjunctivitis	10	4
Chest pain	121	47
Rapid respiration	132	51
Recent loss of hearing	4	2
Bleeding	4	2

#### Anti-Dengue virus antibodies

The prevalence of Dengue specific antibodies (IgM and IgG) was 69.23% and of this 66.15% (172 subjects) were positives for Dengue IgG antibodies. Among the Dengue IgG positives, 66.28% in females and 33.72% in males. There was no statistical difference between Dengue IgG prevalence and gender (p = 0.427) (Table 2). Patients within the age group 21–30 years were observed to have the highest Dengue IgG prevalence of 65.84% (54/82). There was significant association between Dengue IgG seropositivity and age groups (p = 0.01). Serum from subjects within the age group of 5–14 years were not found to be positive for Dengue IgG antibodies.

**Table 2:** Association of demographics and Dengue seropositivity

Characteristics	Total number of patients	Dengue virus IgM	p value	Dengue virus IgG	p value	IgM and IgG
Overall total	260	39		172		31
Sex						
Male	84	13	0.236	58	0.427	10
Female	176	26		114		21
Age						
< 5 years	3	0	0.289	0	< 0.001	0
5–14 years	11	1		0		0
15–20 years	14	5		6		4
21–30 years	82	12		54		8
31–40 years	73	8		45		7
41–50 years	27	8		23		7
51–60 years	22	1		18		1
61–70 years	12	0		11		0
> 70 years	9	2		8		2
Unknown	7	2		7		2

Anti-Dengue virus IgM antibodies accounted for 15% (39/260) of the total patient’s samples tested. Of these, 8 were positive for only Dengue-specific IgM and 31 for both IgM and IgG. A larger proportion of positives, 25% (8/31) for both IgG and IgM were within the age group of 21–40 years compared with the least in the age groups of the ≤ 5 and ≥ 51 years. Males as against females recorded the highest occurrence of Dengue IgM antibodies of 15.48% (13/84) and 14.77% (26/176) respectively, though no significant association was observed for IgM antibodies and gender.

#### Anti-Chikungunya virus antibodies

Total Chikungunya virus antibodies detected was 27.69%. Of this 20.69% were positive for only IgG antibodies. The IgG frequency was higher in males 21.4% (18/84) than in females 20.69% (36/176) though there was no association between gender and IgG seropositivity. Whiles sero-prevalence was higher in the 21–40-year age group (31/54), the age groups within the bracket of ≤ 5 and ≥ 51 years were not found to be positive for anti-Chikungunya IgG antibodies and no association between age and IgG seropositivity (*p *= 0.096) was established.

The occurrence of anti-Chikungunya IgM antibodies was 10.39% and was higher in females, 10.80% (19/176) than males 9.52% (8/84) with no association between gender and IgM seropositivity (*p *= 0.924). Though there was no statistical significance between age and IgM seropositivity (*p* = 0.508), subjects within the age group (21–40) years recorded sero-prevalence of 12.90% more than any other age group.

#### Confirmed Dengue and Chikungunya cases

None of the participants tested positive for Dengue by RT-PCR assay or with the NS1 antigen strip kit. None also tested positive for Chikungunya by RT-PCR.

### Discussion

This study provided evidence that Dengue and Chikungunya viruses are circulating in Ghana, outside of an epidemic. Exposure levels of serum antibodies to these viral pathogens observed suggests transmission intensity.

Dengue specific antibodies (IgM and IgG) was 69.23%. IgM was found positive in 15% (n = 39) and IgG in 66.15% (n = 172). These findings suggest an appreciable level of Dengue fever transmission in urban center of Accra. This observed level of Dengue virus exposure was comparable to that seen in endemic countries in Central America and South-East Asia [13, 14]. Detected IgG antibodies (66.15%) was higher than what was observed in similar studies in Kenya, (12.5%) [15], Sudan (9.4%) [16] and Ghana (21.6%) where the sero-prevalence DENV in malaria positive children was determined [17]. Prevalence of anti-Dengue IgG antibodies was highest among patients in the age bracket of 21–30 years than the other age groups. This finding was consistent with previous studies in Zambia and Burkina Faso [18, 19] but different from the pattern seen in Sudan where children between 5 and 14 years old were found to be the most affected [16].

Chikungunya specific antibodies in this study was 27.69%. IgM was positive in 10.39% (n = 27/260) and IgG in 20.76% (n = 54/260). A similar study in Nigeria and Benin reported 13% and 36.1% of anti-Chikungunya IgG antibodies respectively compared to 20.76% in Ghana [20, 21]. Anti-Chikungunya IgM antibodies of 10.39% in our study may suggest recent infections, notwithstanding, subclinical or possibly apparent that might have been missed and undiagnosed or treated as any common endemic ailment. We observed less prevalence in anti-CHIKV antibodies (27.69%) than anti-DENV (69.23%) which corroborates other seroprevalence studies in Africa [22, 23].

The study recorded no positive signal for RNA detection by real time RT-PCR for Dengue and Chikungunya viruses by the Trioplex real time RT-PCR assay. This contrasts with literature which suggest that the viral RNA to these viruses can be detected from day 0 to day 7 following infection and symptomatology. This could partly be attributed to samples that were collected after the acute phase or onset of illness and therefore chances to detect RNA were limited [12]. The inability to detect viral RNA in our study is corroborated in some studies [24, 25].

Detected levels of anti-Dengue and Chikungunya virus antibodies observed among patients indicates appreciable transmission intensity. The evidence our data provides supports the possible circulation of Dengue and Chikungunya virus which could be causes of subclinical infections or clinical cases but treated for other known endemic conditions.

Increased awareness of these viruses should be built up and differential diagnosis work-up in febrile patients in Ghana should include Dengue and Chikungunya infections. Our study has added to and improved the limited information-base on these viruses in Ghana and will help set up policies and surveillance programs aimed at controlling and prevention and possibly averting outbreaks.

## Limitations

This study data did not compare antibody titers of the acute and convalescent samples from same patients to give a diagnostic indication where there had been a fourfold rise. Additionally, the serology assay for the Dengue virus antibody detection used in this study was not serotype, hence unable to distinguish the circulating serotypes from the antibodies detected.

## Data Availability

In this manuscript, the availability of data and material was not applicable

## Abbreviations

AMED: Japan Agency for Medical Research and Development
DENV: Dengue fever virus
CHIKV: Chikungunya virus
ECSA: east-central-south African
ELISA: enzyme-linked immunosorbent assay
IgG/M: immunoglobulin G/M
J-GRID: Japan Initiative for Global Research Network on Infectious Diseases
NMIMR: Noguchi Memorial Institute for Medical Research
RT-PCR: reverse transcription-polymerase chain reaction
SPSS: Statistical Package for Social Sciences
US CDC: United States of America’s Centers for Disease Control and Prevention
